# Reduced-intensity haploidentical peripheral blood stem cell transplantation using low-dose thymoglobulin for aggressive adult T cell leukemia/lymphoma patients in non-complete remission

**DOI:** 10.1007/s00277-020-03934-6

**Published:** 2020-01-31

**Authors:** Makoto Hirosawa, Takahiro Yamaguchi, Aya Tanaka, Yoshihiko Kominato, Takehiro Higashi, Hiroaki Morimoto, Junichi Tsukada

**Affiliations:** 1 Hematology, University of Occupational and Environmental Health, 1-1 Iseigaoka, Yahatanishi-ku, Kitakyushu, 807-8556 Japan; 2 Department of Legal Medicine, Graduate School of Medicine, Gunma University, Maebashi, Japan

**Keywords:** Haploidentical hematopoietic stem cell transplantation, Adult T cell leukemia/lymphoma, Thymoglobulin and reduced intensity conditioning

## Abstract

Haploidentical hematopoietic stem cell transplantation (haplo-HSCT) has been accepted as a treatment option for aggressive (acute or lymphoma type) adult T cell leukemia/lymphoma (ATLL) patients with a poor prognosis, when a suitable HLA-matched donor is not available. However, haplo-HSCT carries a potential risk of treatment-related mortality including severe graft-versus-host disease (GVHD). Therefore, we conducted a prospective pilot study in order to evaluate the efficacy and safety of reduced-intensity haploidentical peripheral blood stem cell transplantation (haplo-PBSCT) with low-dose thymoglobulin (2.5 mg/kg only on day −2), fludarabine, melphalan, and total body irradiation 4 Gy for aggressive ATLL. Three consecutive acute type ATLL patients, who were ineligible for conventional myeloablative conditioning due to advanced age or comorbidities, were enrolled. One patient received pretransplant mogamulizumab therapy. All the patients were not in complete remission (CR) at the time of transplantation. Our transplantation protocol was safely carried out. CR was achieved in all the patients after transplantation. HTLV-I viral loads became undetectable after transplantation. No severe adverse events such as grade III-IV GVHD or viral/fungal diseases were observed. At a follow-up of 2 years, they were still in CR. However, T cell receptor repertoire diversities were low 1 year after transplantation in next-generation sequencing. Our results show encouraging therapeutic benefits of this pilot approach using reduced-intensity haplo-PBSCT with low-dose thymoglobulin for aggressive ATLL patients.

## Introduction

Adult T cell leukemia/lymphoma (ATLL) is a chemotherapy-resistant malignancy of peripheral T-lymphocytes caused by infection of a retrovirus human T cell lymphotropic/leukemia virus type-1 (HTLV-I) [1–3]. In Japan Clinical Oncology Group Study 9801 (JCOG9801), patients with aggressive (lymphoma or acute type) ATLL showed a poor prognosis with 3**-**year overall survival (OS) of 24% even by using modified LSG15 (mLSG15; VCAP-AMP-VECP) chemotherapy [4]. Moreover, the responses lacked durability and the OS plot for the therapy did not reach a plateau [4]. A nationwide survey of 1594 ATLL patients newly diagnosed from 2000 to 2009 also reported mean survival times (MSTs) of 8.3 months for acute type and 10.6 months for lymphoma type [5]. Despite the application of new agents such as an anti-CCR4 monoclonal antibody mogamulizumab [6], the clinical outcome of aggressive ATLL patients still remains dismal.

Allogeneic hematopoietic stem cell transplantation (allo-HSCT) has been considered as curative treatment modality for patients with aggressive ATLL [5, 7–10]. Approximately 40% of aggressive ATLL patients could survive after allo-HSCT. A graft versus ATLL effect has been demonstrated [8, 9, 11, 12]**.** However, most of the patients with ATLL are over 50 years of age with comorbidities, because the disease develops after a very long latency period of HTLV-I infection. Treatment-related mortality (TRM) after allo-HSCT has been still high. Therefore, it remains uncertain which type of allo-HSCT is suitable for the treatment of aggressive ATLL to minimize disease relapse and early/late complications. Furthermore, this therapeutic strategy has been limited by the availability of a suitable HLA-matched donor.

When an HLA-matched donor is not available, allo-HSCT from haploidentical family donors (haplo-HSCT) may offer the benefit of rapid donor availability. However, information regarding haplo-HSCT for ATLL is so limited that its role and clinical benefits remain unsolved. In this regard, Ogawa et al. reported that a patient with non-CR ATLL achieved CR after transplantation and survived for 382 days in a prospective haploidentical peripheral blood stem cell transplantation (haplo-PBSCT) study with non-myeloablative conditioning using ATG-Fresenius (2 mg/kg for 4 days) [13]. Moreover, we found that unmanipulated haplo-PBSCT following reduced-intensity conditioning (RIC) with low-dose thymoglobulin (2.5 mg/kg only on day −2), fludarabine, melphalan, and total body irradiation (TBI) 4 Gy induced a durable complete remission (CR) without development of severe graft-versus-host disease (GVHD) in a chemo-resistant aggressive ATLL patient [14]. These observations prompted us to conduct a prospective pilot study of unmanipulated haplo-PBSCT using this RIC regimen with low-dose thymoglobulin for aggressive ATLL patients. In the present study, all our patients were in non-CR disease status and were ineligible to myeloablative conditioning (MAC) regimens due to advanced age or comorbidities. As a result, our reduced intensity haplo-PBSCT strategy induced a promising long-term disease-free survival without severe GVHD and infections. To the best of our knowledge, this is the first report of a prospective study for aggressive ATLL patients, which showed the efficacy and safety of unmanipulated haplo-PBSCT following RIC with low-dose thymoglobulin.

## PATIENTS and METHODS

### Patients

Aggressive (acute or lymphoma type) ATLL patients, who were ineligible for conventional MAC due to advanced age (over 50 years of age) or comorbidities, were eligible in the present study. The patients were not in CR at the time of transplantation, and did not have suitable related or unrelated donors within appropriate periods relative to their disease condition. The definition of clinical response to treatment followed the international consensus meeting criteria [15]. Patients were ineligible, if they had severe renal, pulmonary, liver, or cardiac dysfunction. Additional exclusion criteria included left ventricular ejection fraction < 50% in echocardiogram, oxygen saturation < 93% on room air, active serious infection, HIV infection, active central nervous system (CNS) lesions, total bilirubin over 2.0 mg/dl, serum creatinine over 2 times the normal upper limit and allergy to the drug used in this study. Written informed consent was obtained from the patients and their family donors. This protocol was approved by the institutional board of ethics.

### Conditioning regimen and GVHD prophylaxis

**Fig. 1:**
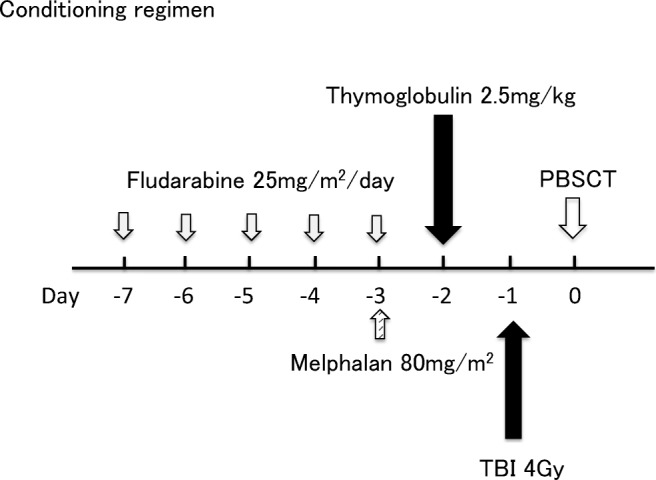
Reduced-intensity conditioning regimen with low-dose thymoglobulin 2.5 mg/kg on day −2, fludarabine 25 mg/m^2^ on days −7 to −3, melphalan 80 mg/m^2^ on day −3 and total body irradiation (TBI) 4 Gy on day −1. Unmanipulated peripheral blood stem cell transplantation (PBSCT) from haploidentical family donor was performed on day 0

Conditioning regimen consisted of low-dose thymoglobulin 2.5 mg/kg on day −2 (day 0 being the first day of donor cell infusion), fludarabine 25 mg/m^2^ on days −7 to −3, melphalan 80 mg/m^2^ on day −3, and TBI 4 Gy on day −1 **(**Fig. 1**)**. The patients received granulocyte-colony stimulating factor (G-CSF)-mobilized unmanipulated PBSCT from haploidentical family donors. GVHD prophylaxis was performed using tacrolimus (target level 10–15 mg/dL) starting on day −1, mycophenolate mofetil (MMF;15 mg/kg twice daily) and methylprednisolone (1 mg/kg). Methylprednisolone and MMF were tapered off until day 30 in the absence of acute GVHD. Tacrolimus tapering was started around day 30 and tacrolimus dose was decreased by 10% every 2 or 4 weeks without evidence of GVHD.

CNS prophylaxis was performed with intrathecal methotrexate before transplantation in order to prevent post-transplant CNS relapse. On the hand, because the national health insurance in Japan has not yet approved anti-viral therapy such as an anti-retroviral nucleotide analog zidovudine for ATLL, our patients did not receive anti-retroviral therapy such as the combination of zidovudine with interferon-α.

### Engraftment and chimerism

Neutrophil engraftment was defined as the first day of 3 consecutive days with an absolute neutrophil count over 0.5X10^9^/L, and the first of 7 consecutive days of transfusion-independent platelet count over 20X10^9^/L was defined as platelet engraftment. Donor chimerism was assessed in whole peripheral blood and bone marrow cells by polymerase chain reaction (PCR) for variable number tandem repeat polymorphisms or fluorescence in situ hybridization (FISH) analysis for the identification of sex chromosomes.

### Supportive care

Patients were treated in high-efficiency particulate-free air room with strict isolation. G-CSF was given from day 5 until neutrophil engraftment. All the patients received anti-bacterial, anti-viral, and anti-fungal prophylaxis with acyclovir, micafungin, and levofloxacin. Trimethoprim/sulfamethoxazole or inhaled pentamidine was given from engraftment. Cytomegalovirus (CMV)-viremia was monitored weekly and ganciclovir or foscarnet was started upon detection of CMV-viremia. Prophylactic antimicrobial therapy continued for patients with active GVHD or those on immunosuppressive treatment.

### Lymphocyte analysis after transplantation

Peripheral blood samples obtained from the patients 1, 3, 6, and 12 months after haplo-PBSCT were analyzed by using flow cytometry to assess post-transplant immune reconstitution. Next-generation sequencing of the T cell receptor β (TCRB) was performed at the Repertoire Genesis Incorporation (RGI; Osaka, Japan) by using the unbiased gene amplification method with Adaptor-Ligation PCR. Bioinformatics analysis was carried out with repertoire analysis software, Repertoire Genesis. Out-of-frame sequences were excluded from the analysis.

## Results

### Patient and donor characteristics

Patient and donor characteristics are shown in Table 1. Three consecutive patients with acute type ATLL aged 53, 64, and 69 years at transplantation were enrolled**.** The patients had good ECOG-performance status (ECOG-PS 0–1) at the diagnosis of ATLL. However, as shown in Table 1, chemotherapy-related toxicities such as sepsis and colitis and/or disease progression worsened their ECOG-PS. At the time of transplantation, ECOG-PS in Cases 1 and 3 was 2. In HTLV-I integration analysis with Southern blotting by *EcoR1* and *Pst1* digestion, Patients 1 and 2 showed defective provirus patterns associated with aggressive ATLL with a poor prognosis [16]. All the patients were treated with intensive chemotherapy mLSG15. In Patient 1, anti-CCR4 monoclonal antibody mogamulizumab (2 cycles, 1 mg/kg, the last infusion on day −76) was combined with mLSG15 regimen. All the patients were not in CR at the time of transplantation. Patients 1 and 2 were in partial remission (PR), and Patient 3 was in progressive disease. At transplantation, the involved lesions were peripheral blood and systemic lymph nodes**.** In Patient 1, mesenterial lesions were also detected**.** The intervals from diagnosis to haplo-PBSCT were 99, 118, and 81 days, respectively. Infused peripheral blood CD34-positive cell counts were 1.93, 4.3, and 3.1 × 10^6^/kg recipient body, respectively. The donors were negative for anti-HTLV-I antibody.

**Table 1:** Patient and donor characteristics

	**Age/ Gender**	**Diagnosis**	**Pre-transplant moga**	**Disease status at HSCT**	**Sites at HSCT**	**ECOG PS** **at diagnosi/at HSCT**	**Interval from diagnosis to HSCT(day)**	**Donor**	**Mismatched HLA**	**CD34+ cell dose/kg recipient body**
**Patient 1**	**53/M**	**Acute type**	**Yes 2 cycles** **until day − 76**	**Partial remission**	**Systemic lymph nodes, Peripheral blood, Mesentery**	**0/2**	**99**	**Niece**	**A,B.C,DR**	**1.93X10**^**6**^
**Patient 2**	**64/F**	**Acute type**	**No**	**Partial remission**	**Systemic lymph nodes, Peripheral blood**	**0/1**	**118**	**Son**	**A,B, DR**	**4.3X10**^**6**^
**Patient 3**	**69/M**	**Acute type**	**No**	**Progressive disease**	**Systemic lymph nodes, Peripheral blood**	**1/2**	**81**	**Son**	**A,B, DR**	**3.1X10**^**6**^

### Transplantation outcomes (Table 2)

At a follow-up of 24, 29, and 28 months, they have been alive and well without relapse. The condition regimen was well tolerated. Rapid hematopoietic engraftment and full donor chimerism on peripheral blood and bone marrow cells were achieved. Neutrophil engraftment was obtained on days 10, 10 and 9 and platelet engraftment on days 10, 13 and 10, respectively. ATLL cells in their peripheral blood disappeared after transplantation. HTLV-I viral load also became undetectable 6 months after transplantation. Patient 1 showed a low HTLV-I viral load 1 year after transplantation, whereas the other two patients were in viral remission 1 year after transplantation. None of the patients received donor lymphocyte infusion after haplo-PBSCT. None of the patients experienced secondary graft failure.

**Table 2:** Transplantation outcomes

**Neutrophil engraftment (day)**	**Platelet engraftment (day)**	**Acute GVHD**	**Extensive chronic GVHD**	**CMV diseas/EBV LPD**	**Disease status after HSCT**	**HTLV-I viral load (copies/1000 PBMCs) at HSCT/6 Mo postHSCT/1 year postHSCT**
**10**	**10**	**II (Skin 2, Gut 1, Liver 0)**	**ー**	**ー/ー**	**CR for 24 Mo**	**658/0/90**
**10**	**13**	**0 (Skin 0, Gut 0, Liver 0)**	**ー**	**ー/ー**	**CR for 29 Mo**	**115/0/0**
**9**	**10**	**I (Skin 2, Gut 0, Liver 0)**	**ー**	**ー/ー**	**CR for 28 Mo**	**257/0/0**

Acute GVHD was tolerable in all the patients. There was one case (Patient 1) with grade II (skin stage 2 and gut stage 1) acute GVHD, who was successfully treated with steroid. In this regard, Patient 1 received mogamulizumab therapy 76 days before haplo-PBSCT. None had extensive chronic GVHD. Tapering-off of immunosuppressive agents was done in all the patients. Transient asymptomatic CMV antigenemia was observed, but none of the patients developed CMV diseases. There was neither Epstein-Barr virus lymphoproliferative disease (EBV-LPD) nor hemorrhagic cystitis. No fungal disease was observed.

### Immune reconstitution after transplantation

**Fig. 2:**
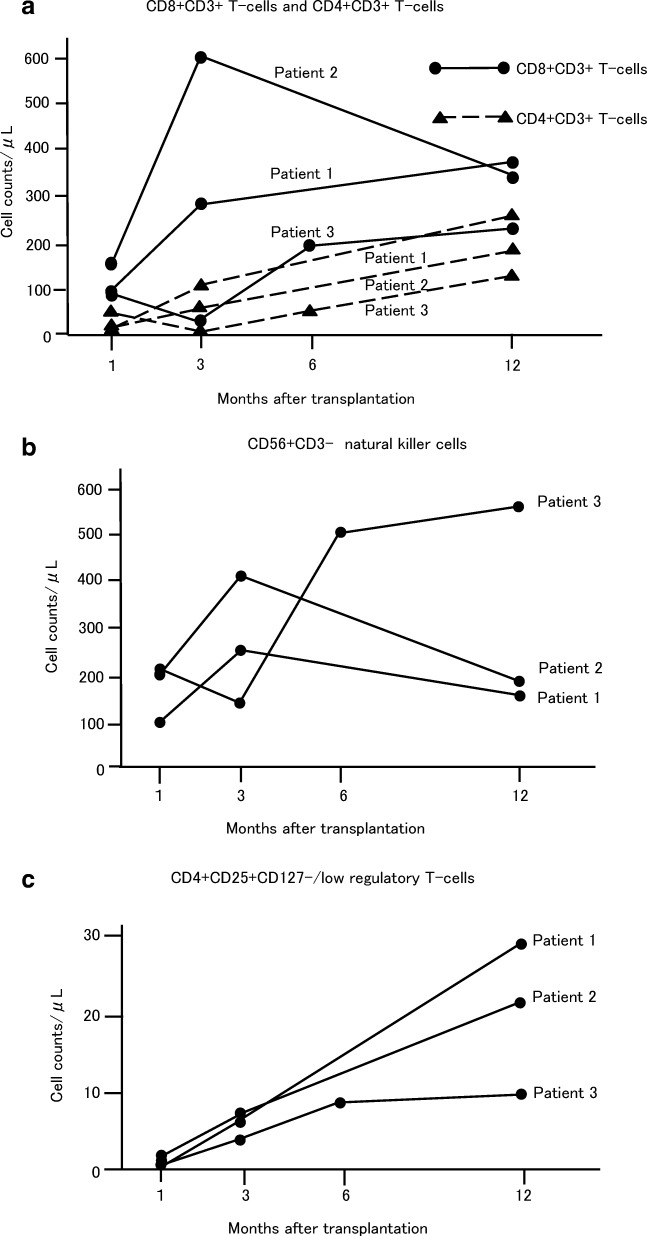
Flow cytometric analysis of lymphocyte reconstitution after transplantation Peripheral blood samples were obtained from the patients 1, 3, 6,and 12 months after transplantation. Numbers of CD8 + CD3 + T-cell (A; closed circles and solid lines), CD4 + CD3 + T-cells (A; closed triangles and dashed lines), CD56 + CD3- Natural Killer cells (B) and CD4 + CD25 + CD127−/low regulatory T-cells (C) were shown.

The changes in peripheral blood cell counts of CD4 + CD3 + T cells, CD8 + CD3 + T cell, CD4 + CD25 + CD127−/low regulatory T cells (Treg cells), and CD56 + CD3- Natural Killer (NK) cells at 1, 3, and 12 months after haplo-PBSCT were shown in Fig. 2. Since Patients 3 show a significant delay in recovery of T cells, additional assessment was performed at 6 months post-transplantation. CD4 + CD3 + T cell counts recovered slowly, when compared with CD8 + CD3 + T cell reconstitution **(**Fig. 2A**)**, showing T cell subset recovery in favor of CD8. One month after transplantation, absolute CD4 + CD3 + T cell counts were still below 50/μL, and the CD4 + T cell/CD8 + T cell ratio ranged from 0.11 to 0.51. Inverted CD4/CD8 ratio was continuously observed during the first year post-transplantation.

On the other hand, rapid reconstitution of NK cells was obtained in all the patients with above 100/μL 1 month after transplantation **(**Fig. 2B**)**. Treg cell is a prognostic cellular biomarker for acute GVHD [17, 18]. One month after transplantation, the numbers of Treg cells were significantly low, showing delayed recovery of Treg cells. However, the absolute numbers of Treg cells increased approximately 10 to 20 times 1 year after transplantation **(**Fig. 2C**)**.

VJ combination and complementarity-determining region3 (CDR3) sequence analysis were performed by using TCRB cDNAs synthesized from RNAs isolated from peripheral blood mononuclear cells obtained from the patients 1 year after haplo-PBSCT. We obtained unique CDR3 reads of 13,564 for TCRB. The TCRB repertoire diversities based on the inverse Simpson’s diversity index and the Shannon-Weaver index were low in all the patients, when compared with those in healthy controls **(**Table 3**)**. There were no significant differences in the TCRB repertoire diversity among the patients. TCRBV27/TCRBJ1–6 (CASSLFLSSPLHF; %Reads 13.98%) and TCRBV27/TCRBJ1–5 (CASSGQSNQPQHF; %Reads 13.83%) were highly used in Patients 1 and 3, respectively, whereas Patient 2 showed a high usage in TCRBV7–9/TCRBJ2–1(CASSGSAGGNEGEF; %Reads 13.44%).

**Table 3:** T-cell receptor β (TCRB) repertoire analysis

	**the Shannon-weaver index**	**the inverse Simpson’s index**
**Patient 1**	**4.47**	**27**
**Patient 2**	**4.86**	**30**
**Patient 3**	**4.44**	**28**
**Healthy controls (*n*** **= 18)**	**Mean 9.457 ranged from 7.65 to 10.65**	**Mean 2077 ranged from 41 to 15,193**

## Discussion

Allo-HSCT has been considered as curative treatment modality for aggressive ATLL [5, 7–10]. However, the survival is often compromised by TRM after transplantation. The high incidence of TRM hinders the wide application of allo-HSCT to ATLL patients. Since we used thymoglobulin, it is also possible that its effectiveness in modulating GVHD may be offset by infectious complications.

In the present study, RIC with low-dose thymoglobulin followed by unmanipulated PBSCT from HLA-haploidentical family donors showed significant therapeutic benefits. Rapid engraftment and durable clinical remission were obtained in all the patients. In a prospective study involving 34 patients with high-risk acute leukemia or malignant lymphoma, Ikegami et al. showed that 42.3% of patients with non-CR status survived 1 year after haplo-PBSCT with RIC regimen using ATG-Fresenius (2 mg/kg for 4 days) [19]. They pointed out that non-CR disease status at the time of transplantation was a significant risk factor associated with post-transplant relapse. A nationwide retrospective study on allogeneic bone marrow transplantation (BMT) and PBSCT for 586 ATLL patients in Japan also demonstrated that non-CR ATLL disease status at transplantation was a statistically significant predictor for poor survival [10]. RIC is less cytotoxic and more dependent on donor cellular immune responses in order to eradicate tumor cells, compared with MAC. If graft versus leukemia (GVL) effect appears earlier after transplantation, ATLL disease activity may be controllable even with RIC.

Several previous studies demonstrated GVL effect for ATLL [8, 9, 11, 12]. A long-term follow-up study of allo-HSCT with RIC for ATLL reported 10 long-term survivors (a median of 82 months after transplantation) [9]. Among them, 8 patients were in PR at the time of transplantation. In a patient developed post-transplant relapse in the skin and lymph nodes, the second CR was induced by discontinuation of cyclosporine [9]. A retrospective allo-HSCT analysis of 15 ATLL patients by Shiratori et al. also showed that four patients achieved CR or PR, when calcineurin inhibitors were reduced or abruptly discontinued for disease control [11]. Fukushima et al. reported a retrospective study of myeloablative allo-HSCT for 40 patients with acute/lymphoma type ATLL [8]. In their study, of 10 post-transplant relapse patients, three achieved the second CR by cessation or reduction of immunosuppressive agents. These results were supported by a large retrospective ATLL study, which showed the association of GVHD development with favorable survival without post-transplant disease progression [20]. Thus, GVL effect plays a crucial and beneficial role in the outcome of allo-HSCT for ATLL. In the present study, we utilized a strong allogeneic response of unmanipulated haploidentical grafts in order to prevent disease relapse after transplantation. Unmanipulated haplo-PBSCT as a stem cell source may be a powerful tool for induction of GVL effect.

ATG, in addition to its T cell depleting properties, possess diverse effects on the immune system through affecting various T cell subsets. Low-dose ATGs induce selective depletion of activated T-cells [21] and stimulate the recovery of Treg cells [22]. In addition, less intensive conditioning can reduce inflammatory cytokine release associated with GVHD development. In the present study, although the TCRB repertoire diversities were low even 1 year after haplo-PBSCT, HLA-mismatch in the GVH direction had no impact on the development of GVHD. Thus, RIC with low-dose thymoglobulin might reduce potential GVHD risks in HLA-incompatibility without increasing infection risk.

Anti-CCR4 monoclonal antibody mogamulizumab has been demonstrated to be clinically effective as alone or in combination with multiagent chemotherapies such as mLSG15 regimen. An overall response rate of 50%, including CR rate of 31% has been reported in a multicenter phase II of mogamulizumab monotherapy [23]. However, since CCR4 is expressed on Treg cells as well as ATLL tumor cells, there are concerns regarding an increased risk of severe acute GVHD in ATLL patients treated with pretransplant mogamulizumab. Actually, recent studies reported increased incidence and severity of acute GVHD in ATLL patients treated with mogamulizumab before transplantation [24–27]. However, acute GVHD observed in Patient 1 responded well to steroid therapy.

Undetectable HTLV-I virus load was obtained in all of the patients 6 months after haplo-HSCT. In Patient 1, HTLV-I virus load increased to a carrier level at 1 year after haplo-SCT. However, detectable virus load is not necessarily associated with disease progression in continued clinical remission [9]. In the present study, all the patients have been clinically in CR after haplo-PBSCT.

A recent nationwide study based on retrospective survey data of the Japan Society for Hematopoietic Cell Transplantation (JSHCT) failed to demonstrate the clinical benefits of haplo-HSCT for ATLL [28]. Although 41.3% of the patients received ATG in their study, it has several limitations common among observational retrospective studies. Patient eligibility and protocol for transplantation including stem cell sources (PBSCT or BMT), conditioning regimens (MAC or RIC) and GVHD prophylaxis were determined by the physicians at each institution. They suggested the necessity for further optimization of GVHD prophylaxis with ATG along with a conditioning regimen and donor source (PBSCT vs. BMT) to reduce non-relapse mortality (NRM) and relapse. Especially, in their study, various doses of ATGs (thymoglobulin and ATG-Fresenius) were administered. In this regard, our study was a prospective study of haplo-PBSCT with a RIC regimen, and low-dose (2.5 mg/kg on day-2) of thymoglobulin was only used in order to minimize toxicity and prevent GVHD.

Early application of allo-HSCT to aggressive ATLL patients has been demonstrated to improve survival and lower the incidence of NRM [29]. Late transplant patients have higher risks of GVHD/infection-related NRM. Haploidentical family donors enables us to carry out transplantation for such aggressive ATLL patients in a timely manner, in order to avoid acquired resistance to treatment and severe toxicities resulted from heavily repeated intensive chemotherapy. However, the number of the patients in our study is not enough to make a firm conclusion. Therefore, the results obtained from the present study should be interpreted carefully, and further evaluation to confirm its safety and clinical efficacy is necessary in larger studies with longer follow-up. Our transplantation protocol was carried out safely and may provide a potential therapeutic option for aggressive non-CR ATLL patients, especially who are over 50 years of age with comorbidities.

## Abbreviations

M: Male
F: Female
Moga: Magomulizumab
HSCT: Hematopoietic stem cell transplantation
ECOG: The Eastern Clinical Oncology Group
PS: Performance status at diagnosis at diagnosis of ATLL
GVHD: Graft versus host disease
CMV: Cytomegalovirus
EBV LPD: Epstein-Barr virus-associated lymphoproliferative disorders
HSCT: Hematopoietic stem cell transplantation
PBMC: Peripheral blood mononuclear cell
CR: Complete remission
Mo: Months
